# Comments on “The possible pathogenesis of macular caldera in patients with North Carolina macular dystrophy”

**DOI:** 10.1186/s12886-023-03100-2

**Published:** 2023-09-08

**Authors:** Kent W. Small

**Affiliations:** Molecular Insight Research Foundation, 411 N. Central Ave. Suite 115, Glendale, CA 91203 USA

I would like to first congratulate the authors, Zhu et al for their interest and work on North Carolina Macular Dystrophy (NCMD). By finding a family with this disease in China, not connected to North Carolina, the authors have again demonstrated what an inaccurate and misleading misnomer North Carolina Macular Dystrophy (NCMD) is. Many inaccuracies, such as natural history, names, diagnoses have been perpetuated in the literature regarding NCMD [1–16]. Some issues, clarifications, and corrections are important to address in this paper by Zhu et al.

The authors state, “Three subtypes of this disease have been described: MCDR1, MCDR2 and MCDR3 [1, 2]”. MCDR1 is the designation given by the Human Genome Organization for the linkage locus of North Carolina Macular Dystrophy as reported by Small et al. 1991. MC = MaCula, D = Dystrophy, R = Retina to distinguish it from Macular Corneal Dystrophy, and 1 = the 1st macular disease mapped in the Human Genome. MCDR2 (Macular Dystrophy, Retinal 2) is a pattern dystrophy with PROM1 (gene designation Promonin-1 gene) mutations, mostly with a bull’s eye appearance and markedly depressed ERGs therefore showing little phenotypic overlap with MCDR1 and MCDR3 (Macular Dystrophy, Retinal 3, the third macular disease mapped). MCDR1 and MCDR3 have identical phenotypes. Referencing MCDR2 in a paper on NCMD has little value and may only confuse readers further.

The authors state, “MCDR1 has been the most intensively analyzed, with the disorder being mapped to chromosome 16q16 in multiple families [3–5]”. This is an error by Zhu et al. Chromosome 16q16 needs to be corrected to 6q16. The author’s selection and omission of references is flawed. Their reference 3 by Small et al. does not even have genetic linkage mapping involved and should not have been referenced here. There are now many publications showing genetic linkage of an NCMD phenotype to chromosome 6, only 2 of which the authors reference. This statement suggests that their references 3–5 are equivalent in significance. The first linkage mapping of a disease is orders of magnitude more difficult than all of the subsequent linkage studies. All subsequent studies are merely confirmatory. Having said that, please note that the initial linkage mapping of NCMD was by Small et al. in 1991 and was the first macular disease mapped in the Human Genome Project hence the designation MCDR1 [7]. Additionally, the authors failed to reference the many additional families with NCMD subsequently linked to the MCDR1 locus. Many of these by Small et al. [8–21].

The authors state, “There have been few reports of NCMD in an Asian population [8, 9]”. The authors failed to reference the first Asian family in which a mutation was found. As reported by Small et al., in 2016, a small Chinese family was found to have a single nucleotide variant (SNV) or mutation in the same DNASE1 site as the original NCMD family [20]. The authors did manage to reference the Chinese family with NCMD in which Wu et al. in 2021 found a large duplication in the MCDR1 locus involving PRDM13. Indeed, the family reported herein by Zhu et al. may have the same duplication and may be a genealogical branch of the family reported by Wu et al. However, we will never know until Zhu et al. define the boundaries of their duplication.

The authors state that they performed “target region sequencing and high-throughput sequencing.” The authors could be more specific about which targeted region they sequenced and their method. Had the authors performed whole genome sequencing (WGS), the boundaries of their duplication could have been defined. This would be helpful information still.

The authors state that several of the family members “had poor vision”. However, later in their manuscript the authors reveal that the patient’s visual acuity was actually 20/63. Most clinicians and patients would not consider 20/63 to be “poor vision.” Most patients with NCMD have reasonably good to sub-normal vision [1, 4–21].

The authors mention, “PRDM13 is a member of a large family of ‘helix-loophelix’ DNV-binding proteins that play an important role in cell differentiation by regulating gene expression during development [13].” The author’s choice of references is inadequate. This reference is mostly about PRDM in general with very little information about PRDM13 specifically. The publication by Watanabe et al. is much more useful and specific to PRDM13 and should have been referenced by the authors [22].

The authors, several times, make reference to “Kent et al.” There is no “Kent et al.” in their reference list and I suspect the authors meant to say Small et al. instead as “Kent” is my first name and is not appropriate to use as the reference.

The authors state, “the diagnosis of NCMD only based on clinical features may lead to misdiagnosis of NCMD [7, 9]”. Again the author’s choice of references is inadequate as there are many more publications with much more useful and directed data regarding the diagnosis and misdiagnosis of NCMD. Additional references should have been included here such as Small et al., Small et al., Small et al., Small et al., Small et al. [4–21, 23].

The authors make the statement “Genetic counseling can not only assist the diagnosis of the proband, but also identify the type of NCMD and the underlying genetic cause of the disease through whole-genome sequencing, thus providing the possibility for further treatment [2, 9]” First, the authors recommend WGS when even they did not do this in their study. This is why the boundaries of their duplication are not known. Second, Bakall, Small et al. showed that intravitreal anti-VEGF (vascular endothelial growth factor) injections can be a useful treatment for the complication of choroidal neovascularization which can occur [24]. Treating the underlying genetic cause is more challenging than the authors have speculated. Their references regarding treatment by Audere et al. and Wu are again flawed and do not even address treatment issues. Additionally, because NCMD is a congenital disease, the “treatment” would have to take place in utero on the developing embryo [20, 21].

The authors state that “P1 is the proband, a boy who was noted to have a mottled macular pigment 10 days after birth.” The fundus photos clearly show a discrete area of absent RPE / pigment which many clinicians would characterize as “atrophy.” The term “atrophy” implies that tissue was once present, but now is no longer present due to some degenerative process / apoptosis etc. NCMD has been well documented now as a congenital hypoplasia / dysplasia of the macula rather than an atrophic process [1, 18, 20, 21]. The authors actually demonstrated this in their 3 month old affected patient, P1. Like many of the names and terms used in NCMD over the last 50 years, atrophy is yet another one that should not be used here. Indeed NCMD is itself a misnomer as suggested by Small et all and as demonstrated with this disease in China [1–6]_._

Lastly, the authors call the grade 3 lesions a “caldera” in the title and throughout the manuscript. As previously published by Small et al., and the authors failed to reference, this term is probably the least accurate of all of the many incorrect names and terms applied to NCMD [14]. This disease in the single large original family has been given at least 6 different names because of continued misinformation in the literature [1, 5, 6]. Continued use of the term “caldera” in this disease further perpetuates this misinformation.

## Data Availability

Not applicable.
